# Hydromethylthionine sustains truncated tau‐dependent inflammation‐lowering effects in mouse brain

**DOI:** 10.1111/febs.70021

**Published:** 2025-02-17

**Authors:** Renato X. Santos, Sophie H. Lee, Richard Lofthouse, Valeria Melis, Lianne Robinson, Michael Leith, Eline Dreesen, Paul Armstrong, Thomas Vorley, Elizabeth A. Goatman, Claire Hull, Gernot Riedel, Claude M. Wischik, Charles R. Harrington

**Affiliations:** ^1^ Institute of Medical Sciences, School of Medicine, Medical Sciences and Nutrition University of Aberdeen UK; ^2^ Scottish Biologics Facility University of Aberdeen UK; ^3^ Department of Chemistry, School of Natural and Computing Sciences University of Aberdeen UK; ^4^ TauRx Therapeutics Ltd. Aberdeen UK

**Keywords:** Alzheimer's disease, frontotemporal dementia, hydromethylthionine, neuroinflammation, Tau protein

## Abstract

Tauopathies are a heterogeneous mixture of neurodegenerative disorders, including Alzheimer's disease and frontotemporal dementia (FTD), characterised by the accumulation of tau filaments in brain tissue. Tau protein aggregation is inhibited by hydromethylthionine (HMT), an effect that appeared to be prevented in clinical trials for subjects already receiving acetylcholinesterase inhibitors or memantine. Since neuroinflammatory responses are associated with tauopathies, we investigated the effect of HMT on the brain immune response and inflammatory status in line 66 (L66) mice, an FTD‐like model overexpressing human tau, in the presence of memantine. We determined whether HMT (5 and 15 mg·kg^−1^), either singly or combined with memantine (20 mg·kg^−1^), would have a sustained impact on neuroinflammation following the cessation of drug administration. The levels of core tau fragments in L66^+/−^ mice (P301S/G335D‐hTau) were decreased in a dose‐dependent manner 12 weeks after the last administration of HMT, an effect that was not affected by memantine. HMT lowered the levels of tumour necrosis factor alpha (TNF‐α), thus favouring an environment conducive to neuronal protection and repair. HMT sustained increased microglial reactivity after its discontinuation, which may assist in the removal of tau aggregates, but co‐administration with memantine prevented the HMT‐sustained activation of microglia. These findings indicate that HMT has a beneficial effect in reducing neuroinflammation that accompanies a decrease in the accumulation of truncated tau species and that these benefits are not susceptible to interference by memantine. In turn, the nature of drug interference between HMT and memantine seems to be independent of tau and related to microglia reactivity.

## Graphical abstract

Hydromethylthionine (HMT) is an inhibitor of tau protein aggregation, but one that appears to be affected in subjects already receiving symptomatic treatments for dementia, such as memantine. We found that HMT decreased both tau aggregation and neuroinflammation in transgenic mice in a manner that was unaffected by memantine. HMT‐induced reactivity of microglia, which may favour the clearance of tau aggregates, was impaired by memantine in a tau‐independent manner.

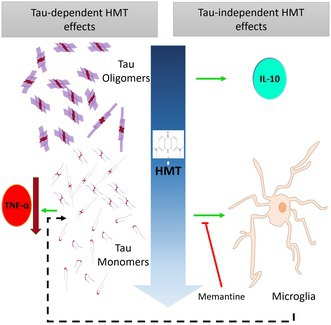

**Abbreviations**
AD: Alzheimer's disease
dGAE: proteolytically stable tau core comprised of amino acid residues Tau297‐391
FL: full‐length
FL‐hTau: full‐length human tau
FL‐mTau: full‐length mouse tau
FTD: frontotemporal dementia
GFAP: glial fibrillary acidic protein
HMT: hydromethylthionine
HMTM: hydromethylthionine mesylate
Iba1: ionised calcium‐binding adaptor molecule 1
IL‐10: interleukin‐10
L66: line 66 FTD‐like mouse model
LC–MS/MS: liquid chromatography‐mass spectrometry
MTC: methylthioninium chloride
NFTs: neurofibrillary tangles
NMRI: Swiss‐type Naval Medical Research Institute mouse wild‐type mouse line
PHFs: paired helical filaments
RT: room temperature
SNARE: SNAP (soluble N‐ethylmaleimide‐sensitive factor attachment) receptors
TAI: tau aggregation inhibitor
TNF‐α: tumour necrosis factor‐alpha

## Introduction

Neuroinflammation is recognised as a hallmark of tauopathies, including frontotemporal dementia (FTD) [1, 2]. Neuropathologically, tauopathies are characterised by the intracellular accumulation of tau, which can be found at different stages of aggregation, from oligomeric species to highly insoluble neurofibrillary tangles (NFTs), consisting of straight and paired helical filaments (PHFs) [3]. The core of the PHF is composed of a truncated 95‐amino‐acid fragment of tau which is capable of catalytic conversion of normally soluble tau into insoluble, intracellular aggregates that accumulate in neuronal cell bodies and dystrophic neurites [4, 5, 6, 7]. Recent advances in the field have shown that pro‐ inflammatory activation of microglia and astrocytes plays a key role as a secondary effector in neurodegenerative cascades in the human brain, and neuroinflammation is considered to precede the onset of symptoms in both Alzheimer's disease (AD) and FTD [8, 9].

Microglial cells are the first line of defence of the brain against insult, being part of the innate immune system of the brain, phagocytosing either pathogens or aberrant deposits of protein aggregates, while triggering a potent inflammatory response [10]. Astrocytes are known to support neuronal function as well as acting as modulators of the inflammatory response [11]. Microglia exist with a wide range of morphologies, motility and ultrastructural features, and with diverse underlying metabolic, proteomic, transcriptomic and epigenetic landscapes that correlate with a multitude of determinants such as species, sex, genetic background, age, spatial location and environment. As a result of such diverse combination of factors determining numerous functional states, microglia are currently considered the most dynamic cells in the healthy mature brain [12]. Consequently, microglia are now better described functionally as homeostatic, when describing basal conditions, and reactive when a specific stimulus is being studied [12]. Although microglia and astrocytes are activated as a defensive mechanism in response to tau pathology, it is the failure to resolve this response that leads to a state of chronic inflammation, which in turn may potentiate tau pathology through mechanisms such as activation of the inflammasome [13, 14]. The chronic reactivity of both microglia and astrocytes contributes to a complex neuroinflammatory response in neurodegenerative disease.

Indeed, clinical studies in FTD and AD have shown positive correlations between both neuroinflammation and the presence of protein aggregates with disease progression, confirming that brain inflammation plays a central role in the progression of neurodegeneration [15, 16, 17]. Post mortem analysis of tissues from AD patients has revealed that astrocyte pathology is not directly correlated with the accumulation of either amyloid‐β or tau proteins, suggesting that other pathological changes may play a role in activating cellular responses [18]. Additionally, alterations in brain and CSF cytokine levels in neurodegenerative diseases, such as AD and FTD, have been reported [19, 20, 21]. Various studies have shed light on the interrelation among tau pathology, neuroglia and neurons at a molecular level. Distinct types of microglia, with specific biochemical signatures and spatial localisation, have been identified as limiting the progression of AD, undergoing a two‐step activation process where the molecular switch seems to be the triggering receptor expressed on myeloid cells 2 (Trem2) [22]. Such a mechanism has been described in both the 5XFAD mouse model and in a mouse model of amyotrophic lateral sclerosis (ALS), suggesting that such a mechanism may be conserved in neurodegeneration [22]. For tau, most *in vivo* studies have been performed using the PS19 mouse model of tauopathy, in which human tau (hTau) carrying the P301S mutation is expressed under control of the mouse prion promoter [23]. The PS19 mice have early synaptic loss and microglial activation, preceding the activation of astrocytes [23], and neurons harbouring tau aggregates expose membrane phosphatidylserine to trigger engulfment by microglia [24].

Hydromethylthionine mesylate (HMTM) is the stabilised anhydrous crystalline dihydromesylate salt that, upon absorption, delivers hydromethylthionine (HMT) to the blood and brain [25, 26]. When referring to the drug, describing the formulation or the administration regime, we have used HMTM. The HMT designation is used when discussing the active moiety of the drug *in vivo* and its proposed mechanism of action. HMT both inhibits tau aggregation and disaggregates tau oligomers and filaments [6, 26, 27]. Two Phase 3 clinical trials have tested HMTM in mild‐to‐moderate AD, and a further trial for behavioural variant FTD (bvFTD) was also conducted with HMTM [28, 29, 30]. In these trials, HMT showed exposure‐dependent pharmacological activity on clinical decline and brain atrophy in AD and FTD at a dose of 8 mg·day^−1^ [30, 31]. Treatment responses to monotherapy were found to be approximately twofold better than those produced by HMT given to participants already receiving treatment with either cholinesterase inhibitors or memantine. The attenuation of the treatment effects of HMT by chronic pre‐treatment with drugs that offer symptomatic benefit was recapitulated in tau transgenic mice as a result of homeostatic down‐regulation of multiple neuronal systems in response to the sustained hyperactivation generated by treatment with standard symptomatic drugs for AD [32, 33]. In addition to its primary pharmacology as a TAI, HMT increases acetylcholine levels in the hippocampus of control mice [32]. Thus, HMT has a dual mechanism of action with the latter being independent of its effect on tau aggregation pathology. It is important, therefore, to establish the long‐term effects of HMTM on different pharmacological targets and determine whether improved neuronal function persists following termination of treatment.

Here, we report that HMT produces a dose‐dependent, sustained reduction in levels of truncated core tau protein in a transgenic mouse tauopathy model (line 66; L66). The L66 mouse expresses hTau protein carrying a P301S/G335D double mutation, with P301S being a mutation that is associated with familial FTD [34]. Single‐point mutations in the tau residue Gly‐335 have been associated with FTD, G335V [35], G335S [36] and G335A [37]. The G335D mutation has not been identified as a disease‐associated mutation, but it was incorporated to enhance tau aggregation. The L66 mouse presents with an FTD‐like motor deficit [38]. Given that a neuroinflammatory phenotype has been reported in a mouse model similar to L66 [39], and that methylthioninium chloride (MTC) ameliorates inflammation in the brain in mice and rats [40, 41], we investigated whether there is any effect of HMTM on inflammatory pathways, either alone or in combination with memantine. HMT triggered a dose‐dependent long‐lasting activation of microglia through a tau‐independent mechanism after cessation of drug administration. HMT also promoted a tau‐related switch‐off of the inflammatory pathways. Our findings demonstrate that the HMTM‐induced cellular immune response was affected by pre‐treatment with memantine.

## Results

### Decreased levels of truncated tau are sustained after a lengthy period of HMT washout

**Fig. 1:**
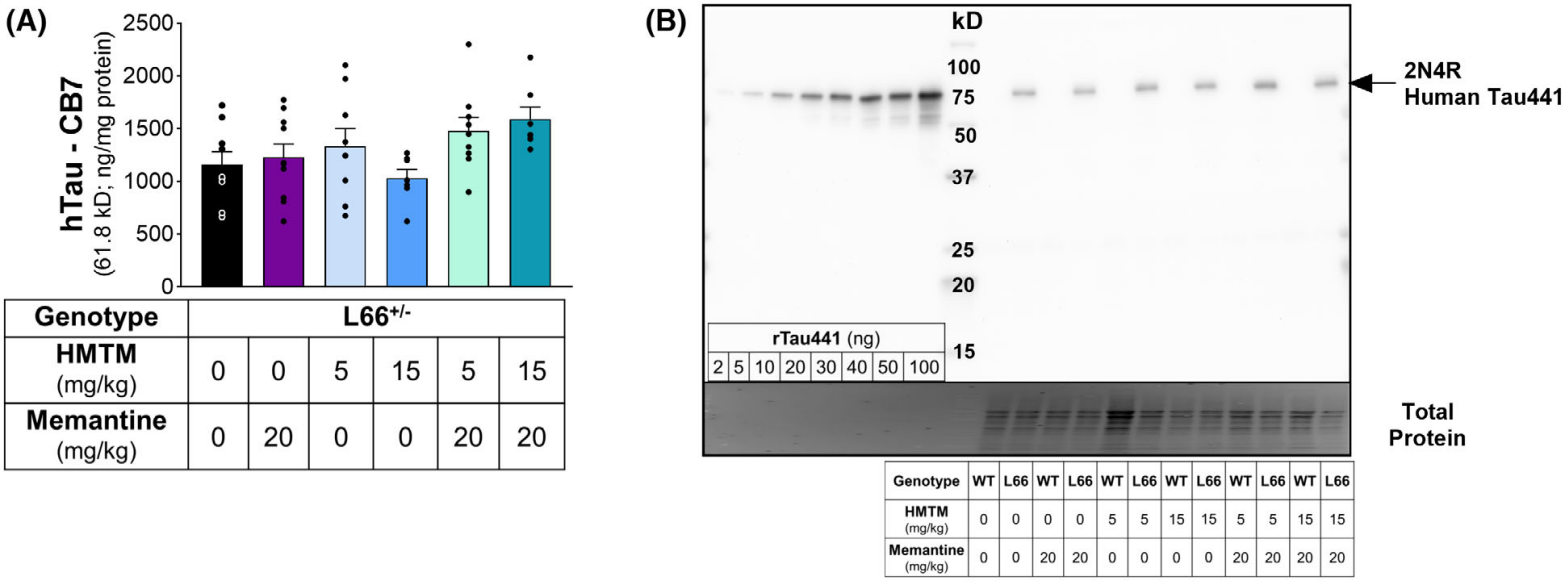
Levels of FL‐hTau in L66^+/−^ mice detected by the antibody CB7 (N‐terminal). CB7 was used to detect FL‐hTau (2N4R; ng·mg^−1^ protein) in the brain homogenate of L66^+/−^ mice dosed with memantine (20 mg·kg^−1^) and HMTM (5 and 15 mg·kg^−1^), either singly or as add‐on, 12 weeks after the cessation of drug administration (A). Representative whole‐membrane immunoblot accompanied by total protein staining (NMRI mice are indicated as wild‐type – WT) (B). Recombinant human tau, isoform 2N4R with 441 amino acid residues, was separated, alongside brain samples, in incremental quantities (2–100 ng), in order to extrapolate the absolute levels of FL‐hTau in the tissue (see Fig. S1A). No differences in the levels of hTau were detected in L66^+/−^ mice under any treatment, following one‐way ANOVA analysis. Data are expressed as mean ± SEM values (*n* = 7–10, details of exact *n* values per experimental group are included in Table 2).

**Fig. 2:**
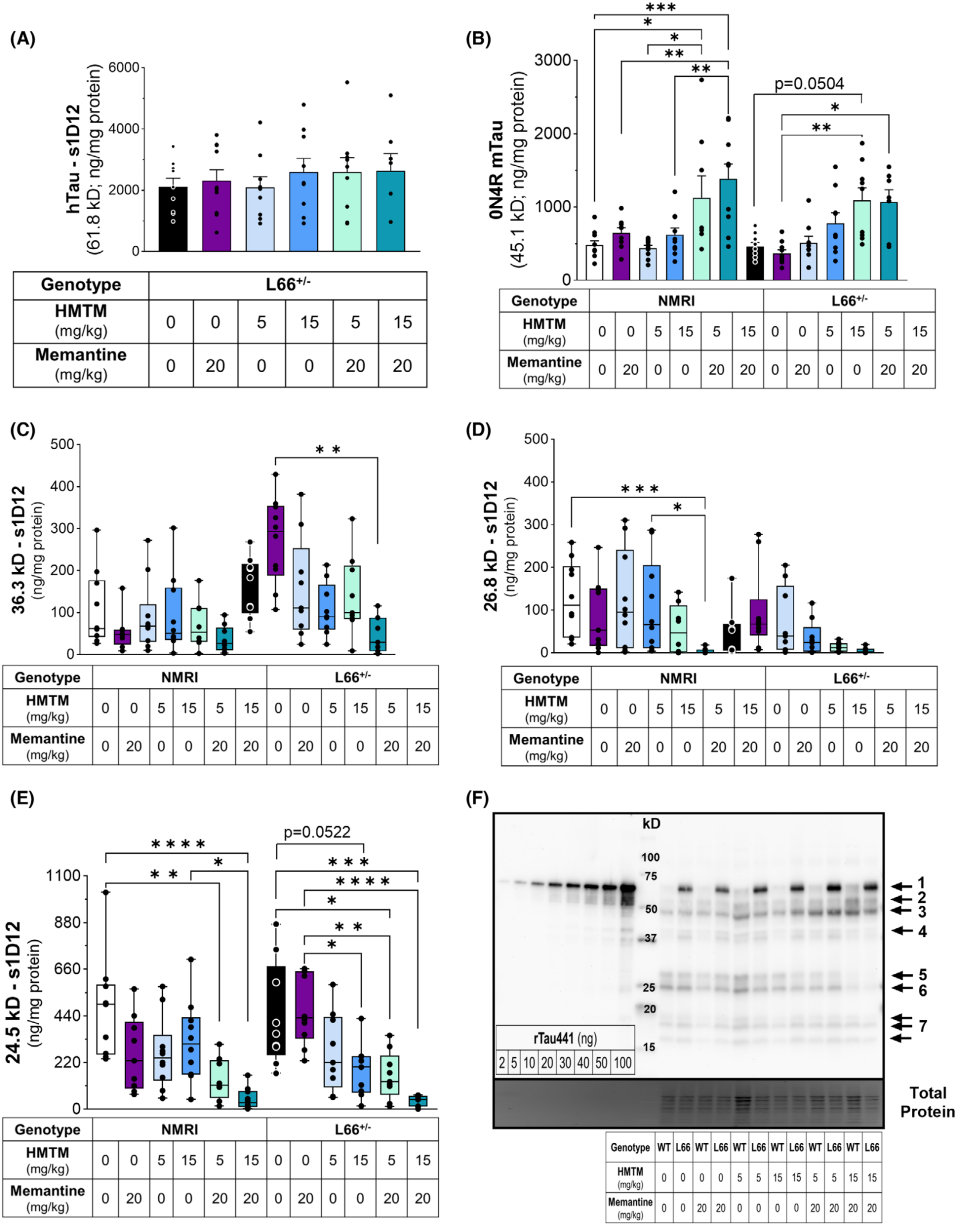
Levels of FL‐Tau and truncated tau fragments in NMRI and L66^+/−^ mice, detected by the antibody s1D12 (repeat domain). Levels of FL‐hTau (A); FL‐mTau (0N4R isoform – most abundant in the adult mouse brain; ng·mg^−1^ protein) (B); the 36.3 kD (ng·mg^−1^ protein) (C), 26.8 kD (ng·mg^−1^ protein) (D) and 24.5 kD (ng·mg^−1^ protein) (E) tau fragments detected by s1D12 in the brain homogenates of NMRI and L66^+/−^ mice dosed with memantine (20 mg·kg^−1^) and HMTM (5 and 15 mg·kg^−1^), either singly or as add‐on, 12 weeks after the cessation of drug administration. Representative whole‐membrane immunoblot showing the 2N4R FL‐hTau (1), 1N4R (2) and 0N4R (3) FL‐mTau isoforms, a 36.3 kD doublet (4), 26.8 kD (5) and 24.5 kD (6) fragment bands as well as a 15–20 kD triplet (7), accompanied by total protein staining (NMRI mice are indicated as wild‐type – WT) (F). Recombinant human tau, isoform 2N4R with 441‐amino‐acid residues, was separated, alongside brain samples, in incremental quantities (2–100 ng), in order to extrapolate the absolute levels of FL and truncated tau in the tissue (see Fig. S1B). Memantine and HMTM administered in combination trigger a sustained increase in the levels of FL‐mTau in both NMRI and L66^+/−^ mice. Data are expressed as mean ± SEM values (A, B) or as median ± IQR (C–E) (*n* = 7–10, details of exact *n* values per experimental group are included in Table 2). A one‐way ANOVA was used to analyse (A, B) and a Kruskal–Wallis test to analyse (C–E). Statistical differences: *P < 0.05; **P < 0.01; ***P < 0.001; ****P < 0.0001.

The levels of FL‐hTau in L66^+/−^ mice 12 weeks after the last dosing of either or both memantine and HMTM, detected by either N‐terminal (CB7; Fig. 1) or core‐specific (s1D12; Fig. 2) antibodies, were similar to vehicle‐dosed L66^+/−^ mice, suggesting no drug effects in FL‐hTau (Fig. 1A: hTau CB7 – *F*(5, 45) = 2.233, *P* = 0.0673; and Fig. 2A: hTau s1D12 – *F*(5, 45) = 0.3607, *P* = 0.8727). In contrast, the levels of the most abundant endogenous isoform of FL‐mTau in mice, detected by s1D12, were increased following administration of a combination of memantine and HMTM, for both NMRI and L66^+/−^ mice (Fig. 2B). Immunoblot analysis revealed that s1D12 (tau 341–353) not only detected full‐length (FL)‐murine tau (mTau) and hTau but also detected fragments at lower molecular weights (triplet: 15–20 kD; 24.5 kD; 26.8 kD; duplet: 36.3 kD) (Fig. 2C–E).

**Fig. 3:**
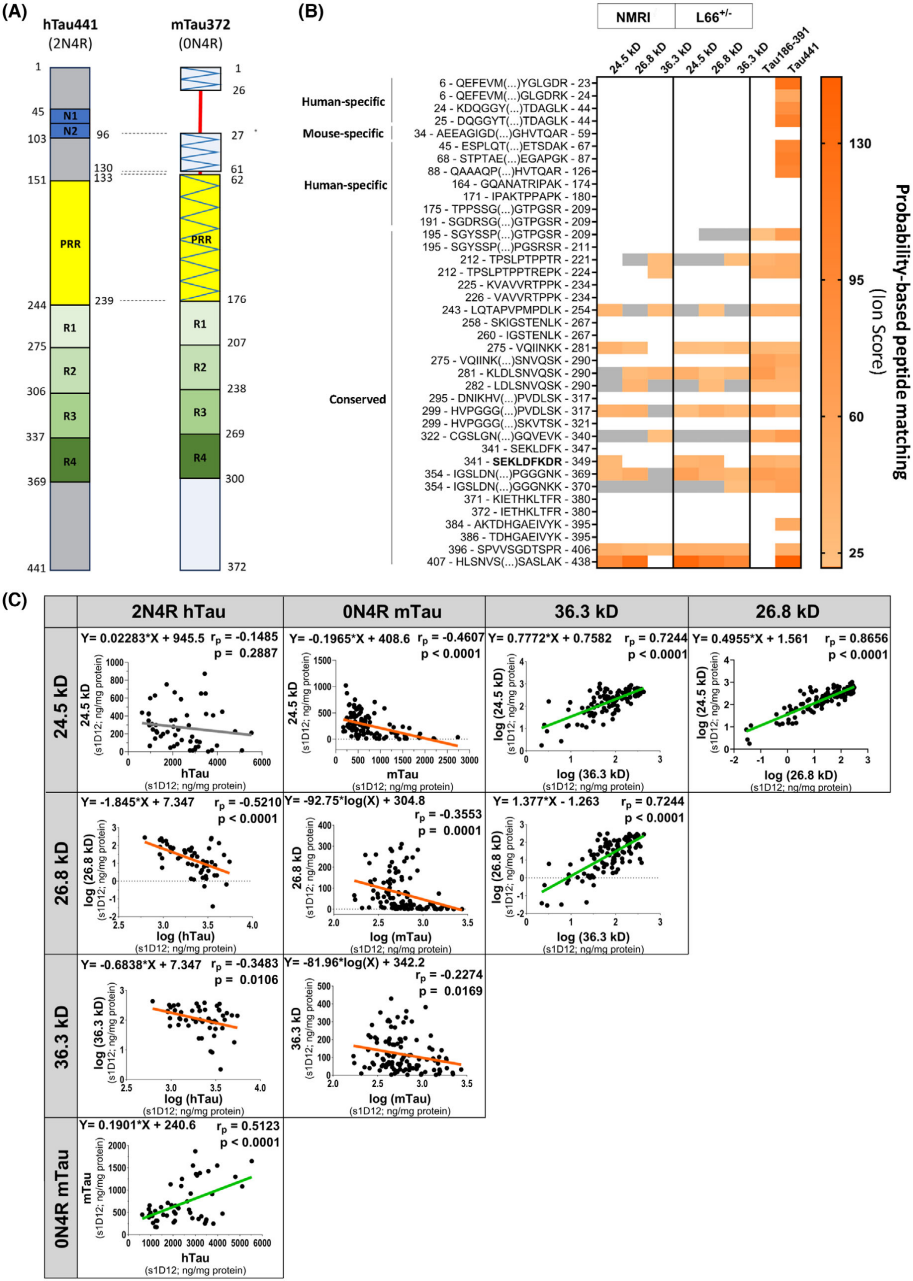
Peptide sequencing and identification of the s1D12‐labelled bands at 36.3, 26.8 and 24.5 kD as truncated tau fragments. Heatmap representing the LC‐MS/MS ion scores for the resulting in‐gel trypsin digestion peptides of the truncated tau species in the bands at 36.3, 26.8 and 24.5 kD labelled by s1D12. A total of 39 peptides, spanning the N‐terminus (N1 and N2), proline‐rich region (PRR), repeat domains (R1‐4) and C‐terminus, from *in silico* predicted trypsin digestion of the 2N4R isoform of human tau are indicated in the heatmap. The depictions of the distinct biochemical regions of both 2N4R human and 0N4R mouse tau isoforms (the most abundant isoforms in adult brains) are represented for an easy visualisation of which peptides are human specific, with the respective amino acid residue alignment between both isoforms. The hatched pattern in the mouse tau indicates regions containing amino acids that are not conserved in the corresponding human tau regions (A). Database search peptide matches with false discovery rate above 5% (FDR > 5%) were considered low‐confidence protein hits (grey in the heatmap), while 1% ≤ FDR ≤ 5% and FDR > 5% were considered medium‐ and high‐confidence hits (orange gradient in the heatmap), respectively. The higher the ions score, the higher the confidence that the spectral scan of the peptide can reliably be matched to the predicted product of cleavage of tau by trypsin. Ion scores below 21 corresponded to low‐confidence protein hits (Table S1). Recombinant protein controls (Tau181–396 and hTau 441) were included (B). The ion scores shown represent the result of the mean of two mice (*n* = 2) for each genotype (NMRI and L66^+/−^). The highlighted peptide spanning amino acid residues 341–349 encompasses the epitope of the antibody s1D12 (amino acids 341–353) used to immunopurify the samples. Correlogram of levels of FL‐Tau and truncated tau species in the bands at 36.3, 26.8 and 24.5 kD labelled by s1D12 (C). The correlation coefficient of Pearson (*r*_P_) is shown along with the corresponding *P*‐values. Inverse correlations and linear rates of decline are shown in orange and direct correlations and respective rates of increase are shown in green. Linear regression lines shown in grey represent a lack of correlation between variables. The study found an inverse correlation between human and mouse full‐length tau isoforms, supporting the origin of truncated tau sequences, and found no significant correlation of the 24.5 kD band with full‐length human tau, but observed positive correlations with a number of tau fragments. *P*‐values were considered statistically significant when *P* < 0.05.

To confirm that the lower‐molecular‐weight fragments were products of truncation of the FL‐Tau protein, tau proteins were immunoprecipitated from NMRI and L66^+/−^ brain samples and analysed by peptide mapping using liquid chromatography–mass spectrometry (LC–MS/MS). It is important to note that identification of all lower MW bands, particularly 15–20 kD, after trypsin digestion, which cleaves proteins at the carboxyl side of arginine and lysine residues, was not possible due to the optimum mass range for LC–MS/MS identification (750–3000 Daltons). Trypsin typically produces peptides with average size of 700–1500 Daltons falling within the ideal range for MS spectrum generation and subsequent peptide fingerprinting [42]. An initial attempt to confirm the identity of the fragments in these bands proved inconclusive (data not shown). The results for the 15–20 kD bands and proteolytically stable tau core comprised of amino acid residues Tau297‐391 (dGAE; part of the proteolytically stable core of the paired helical filaments with self‐aggregating properties) were not totally unexpected for two main reasons: (a) the low levels of protein fragment in these samples (Fig. 2F); and (b) the high density of lysine residues in the core region of tau (dGAE represents 21.5% of the total hTau441 sequence, yet it contains nearly 32% of all lysine residues in the protein sequence), likely leading to the generation of tryptic peptides that were too small to be included in the ideal MS range. Thus, we focused on the identification of the tau fragments detected between 24.5 and 36.3 kD. Among the protein hits with higher peptide spectrum match were several human keratin isoforms (Table S1). It is known that keratin isoforms are common protein contaminants detected in mass spectrometry samples, from hair, skin and reagents [43]. Based on the ion scores from a peptide match search of a database comprising the UniProt human and mouse proteomes, the bands at 24.5, 26.8 and 36.3 kD were identified as being tau fragments based on a total of 23 digestion peptides. Twelve of these peptides were unique and the other 11 shared with other protein groups, with a mean total protein coverage of 67% relative to the 2N4R hTau isoform (Table S1). These sequences spanned from amino acids 195 to 212–438 (2N4R hTau sequence) and/or 126 to 143–369 (0N4R mouse tau sequence). Thus, they encompassed sequences spanning half of the proline‐rich region to the C‐terminal end of the protein (Fig. 3A,B; Table S1). Invariably, the N‐terminal fragments of the tau sequence were not detected in the bands containing truncated tau species which, at least for L66^+/−^ mice, is consistent with the absence of labelling with CB7 (human‐specific N‐terminal antibody) (Figs 1 and 3B). Due to the highly conserved nature of the repeat region, it was not possible to determine whether there was a differential contribution coming from the human or mouse sequences of the FL‐tau. However, correlation analysis from the immunoblotting data shows a significant inverse correlation between the levels of human and mouse FL‐tau isoforms with the levels of the lower‐molecular‐weight fragments further confirming the origin of the truncated forms from the FL‐tau sequences (Fig. 3C). Interestingly, there was one exception to this overall observation; the band at 24.5 kD did not show a significant correlation with the levels of FL‐hTau (Fig. 3C). As expected, the abundance of the truncated tau correlated positively among the different fragments (Fig. 3C), which could reflect the self‐propagating pathological process of truncation and aggregation of the protein, characteristic of the region of the protein contained within tau (297–391) [44].

Whereas memantine alone did not seem to sustain any differences in the levels of truncated tau, it did not prevent HMT from triggering a dose‐dependent decrease in the levels of the 36.3 and 24.5 kD truncated tau in L66^+/−^ mice (Fig. 2C,E). Interestingly, the effects of the administration of the drugs are similar in NMRI and L66^+/−^ mice for the levels of tau at 24.5 kD, but not for the 36.3 kD fragments (Fig. 2C,E), implying that endogenous mTau contributes to the 24.5 kD fragments.

**Fig. 4:**
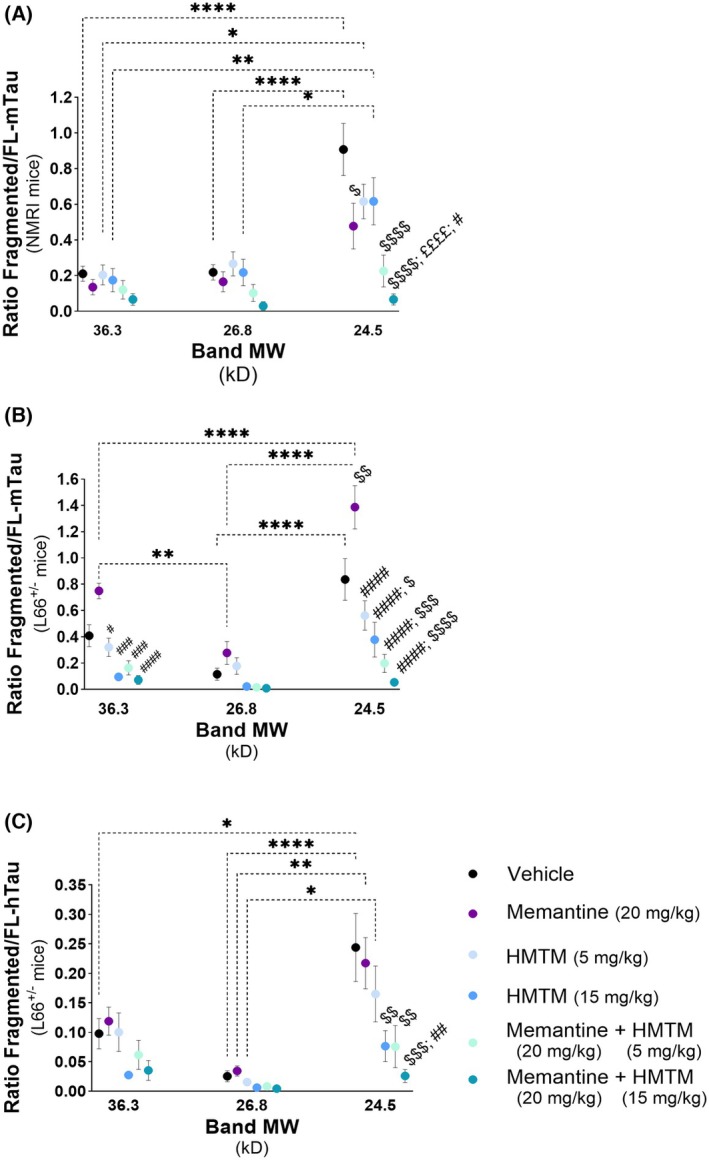
Normalised tau fragment quantification by FL‐mTau and ‐hTau. Ratios of tau fragments (36.6, 26.8 and 24.5 kD) to FL‐mTau in NMRI mice (A), FL‐mTau in L66^+/−^ mice (B) and FL‐hTau in L66^+/−^ mice (C). In NMRI mice, normalised levels of 24.5 kD fragments were significantly decreased by memantine alone, and HMTM (5 and 15 mg·kg^−1^) as an add‐on. In L66^+/−^ mice, 24.5 kD fragments normalised to FL‐mTau increased with memantine compared to vehicle but showed no change when normalised to FL‐hTau. However, memantine did not interact with the ability of HMTM, particularly at 15 mg·kg^−1^, to induce a long‐term decrease in the 24.5 kD fragment. Significant treatment differences observed only in L66^+/−^ mice occurred in the 36.3 kD fragment normalised with FL‐mTau where HMTM, either singly or as add‐on, produced an opposite and significant decrease of the 36.3 kD fragment in relation to memantine. Data are expressed as mean ± SEM values (*n* = 7–10). Treatment differences between band sizes are represented by dashed lines. Within the same band size, treatment differences are indicated by specific symbols: £ denotes a significant difference compared to the 15 mg·day^−1^ treatment group, $ indicates a significant difference compared to the vehicle control and # represents a significant difference compared to the memantine group. Data are expressed as mean ± SEM values (*n* = 7–10, details of exact *n* values per experimental group are included in Table 2). A two‐way ANOVA was used to analyse the data. Statistical differences: *P < 0.05; **P < 0.01; ***P < 0.001; ****P < 0.0001. *P*‐values were considered statistically significant when *P* < 0.05.

To further elucidate the effects of drug administration on the levels of truncated tau after an extensive period of drug washout, we then controlled for the variation in levels of both mouse (Fig. 4A,B) and human (Fig. 4C) FL‐tau, by determining the ratio of tau fragments to FL species. The 24.5 kD fragments were more abundant than the 26.8 and 36.3 kD fragments, in both NMRI and L66^+/−^ mice, and it was also the fragment size in both mouse models that showed more prominent treatment‐dependent variations, reflected in an interaction between fragment size × treatment (Fig. 4A: *F*(10, 151) = 3.272, *P* < 0.001; Fig. 4B: *F*(10, 134) = 5.237, *P* < 0.0001; Fig. 4C: *F*(10, 127) = 1.891, *P* = 0.0522). In NMRI mice, the normalised levels of 24.5 kD fragments were slightly decreased by HMTM (5 and 15 mg·kg^−1^), but significantly decreased when HMTM was administered as add‐on to memantine, and also when memantine was administered alone (Fig. 4A). The smaller effect of HMTM in the presence of memantine in NMRI mice seems to be largely due to the presence of memantine since the combined effect is significantly different from each of the doses of HMTM when administered singly (Fig. 4A). Interestingly, in L66^+/−^ mice, the 24.5 kD band, when normalised to FL‐mTau, was increased by memantine dosing in comparison with the vehicle‐dosed mice (Fig. 4B) and produced no effect when the levels of the 24.5 kD fragments were normalised with FL‐hTau (Fig. 4C). Under both normalisations, memantine in L66^+/−^ mice did not impair the ability of HMTM, particularly at 15 mg·kg^−1^, to induce a long‐term decrease in the levels of 24.5 kD fragments (Fig. 4B,C). In L66^+/−^ mice, a unique observation arose when the levels of 36.3 kD fragments were normalised to the levels of FL‐ mTau. As with the 24.5 kD fragments, memantine promoted an increase of the 36.3 kD truncated tau, whereas they were decreased by the higher dose of HMTM (15 mg·kg^−1^), whether administered singly or as an add‐on (Fig. 4B).

The findings show that there was a continued long‐term effect of HMTM on tau pathology following cessation of treatment. Using data normalised to FL‐tau, it appears that the 24.5 kD truncated tau is a robust indicator of drug efficacy, yet the 36.3 kD fragment may be a more specific marker when investigating drug efficacy in tau transgenic mice. Finally, given the observed correlation between the levels of truncated and FL‐tau, the normalised levels of truncated tau forms may provide a more sensitive indicator of drug efficacy by eliminating individual sample variations.

### HMTM and memantine induce microglial response independent of tau pathology

**Fig. 5:**
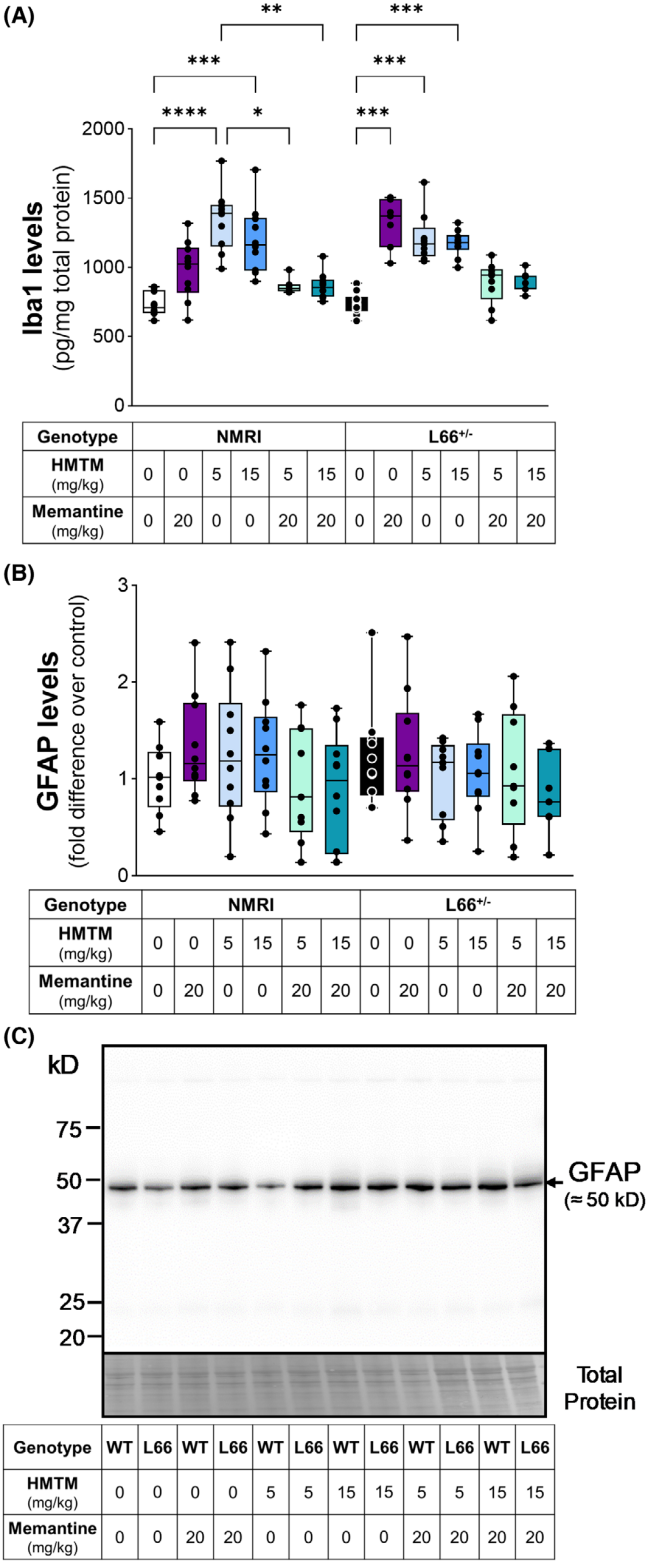
Markers of innate immune cell reactivity in the brain. Levels of the microglial marker Iba1 (A) and the astrocytic GFAP (B) in NMRI and L66^+/−^ mice. Representative whole‐membrane immunoblot showing GFAP staining (NMRI mice are indicated as wild‐type – WT) (C). Both memantine and HMTM monotherapies resulted in a sustained increase in microglial activation (Iba1). However, no changes in GFAP levels across treatment groups or genotypes were observed. Data are expressed as median ± IQR values (*n* = 7–10, details of exact *n* values per experimental group are included in Table 2). A Kruskal–Wallis test was used to analyse (A, B). Statistical differences: *P < 0.05; **P < 0.01; ***P < 0.001; ****P < 0.0001.

The reactivity of microglia is one of the primary immune response events in case of injury or disease. Among many other functions, such as synapse remodelling, vasculogenesis and neurogenesis, microglial cells are critical in neuroinflammation and in the production of cytokines in response to disease processes, such as the accumulation of aberrant protein aggregates [13, 45]. Astrocytes, as well as providing trophic support for neurons, are also part of the CNS immune system, producing cytokines, that act in communication with microglia [46, 47]. While a more detailed analysis and integrative approach would be needed to fully understand the functional role of the innate immune response in the brain to HMTM, we sought to primarily use well‐established protein expression markers of cellular reactivity in this study. To determine changes in reactivity of the CNS cellular immune system, we analysed the levels of the microglial marker ionised calcium‐binding adaptor molecule 1 (Iba1) and the astrocytic glial fibrillary acidic protein (GFAP). For both NMRI and L66^+/−^ mice, administration of either HMTM (5 and 15 mg·kg^−1^) or memantine alone increased levels of Iba1 (Fig. 5A). However, when HMTM was administered as an add‐on to memantine, no increase in Iba1 was observed and the levels remained similar to those observed for vehicle‐treated controls (both NMRI and L66^+/−^), suggesting a negative interaction between the drugs. No changes in GFAP were found in NMRI or L66^+/−^ mice in any treatment group following a prolonged period of drug washout (Fig. 5B). Therefore, these data suggest that either drug caused increased reactivity of microglia when administered singly. The effects of HMTM and memantine in the microglia response were similar for both NMRI and L66^+/−^ mice (Fig. 5A), suggesting a tau‐independent effect, which is further supported by the absence of a correlation between the levels of Iba1 and either s1D12‐reactive FL‐tau (human and murine) or tau fragments (36.3, 26.8 and 24.5 kD; Table 1).

**Table 1:** Correlation between levels of Iba1 and both FL and truncated tau fragments. There were no significant correlations between the levels of Iba1 and the levels of s1D12‐reactive FL‐tau (human and murine) and tau fragments. All values were transformed into *z*‐scores.

Iba1 *vs*:	hTau (CB7)	hTau (s1D12)	mTau (s1D12)	36.3 kD (s1D12)	26.8 kD (s1D12)	24.5 kD (s1D12)
*r*	0.1872	0.0168	−0.0658	0.1267	0.0148	−0.1423
95% CI	−0.0962, 0.4425	−0.2628, 0.2938	−0.2534, 0.1265	−0.0656, 0.3100	−0.1765, 0.2050	−0.3242, 0.0498
*P*‐value	0.1930	0.9077	0.5027	0.1956	0.8804	0.1457

### HMTM promotes a long‐lasting lowering of inflammation after washout

**Fig. 6:**
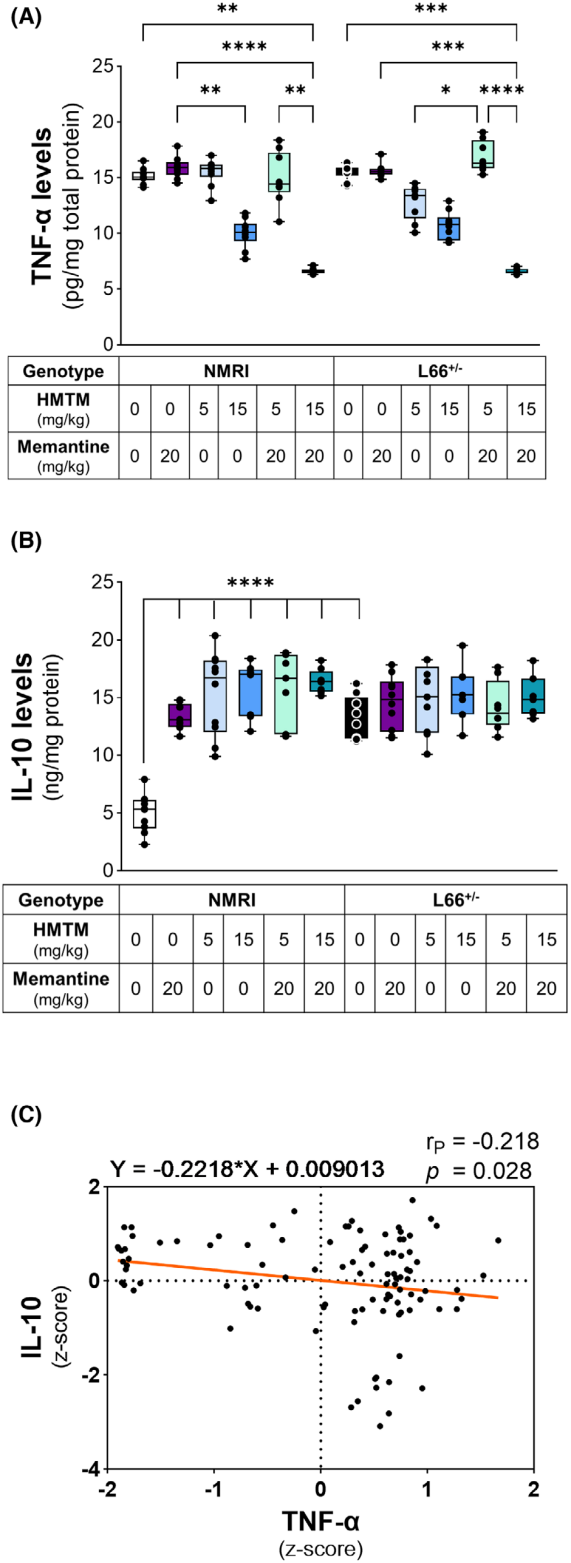
Levels of inflammatory markers in the brain. TNF‐α (A) and IL‐10 (B) were quantified in NMRI and L66^+/−^ mice. HMTM produced a dose‐dependent decrease in TNF‐α levels in both NMRI and L66^+/−^ mice, both as monotherapy and in combination with memantine, compared to vehicle‐treated controls. Neither memantine nor 5 mg·kg^−1^ HMTM (alone or in combination) altered TNF‐α levels in either genotype. IL‐10 levels remained unchanged in L66^+/−^ mice across treatments but were significantly elevated in vehicle‐treated L66^+/−^ mice compared to NMRI mice. In NMRI mice, both memantine and HMTM (5 and 15 mg·kg^−1^) increased IL‐10 levels, with this increase remaining unaffected by combination treatment. Data are expressed as median ± IQR values (*n* = 7–10, details of exact *n* values per experimental group are included in Table 2). Correlation between the levels of IL‐10 and TNF‐α (C). Data show significant inverse correlation between the levels of TNF‐α and IL‐10 after a long washout period. All values were transformed into *z*‐scores. A Kruskal–Wallis test was used to analyse (A, B). Statistical differences: *P < 0.05; **P < 0.01; ***P < 0.001; ****P < 0.0001.

Chronic inflammation can result in tissue damage. Hence, we examined the levels of TNF‐α and IL‐10, as key cytokines, to assess the inflammatory status within L66^+/−^ brains (Fig. 6). HMTM produced a dose‐dependent (15 mg·kg^−1^) decrease in the levels of TNF‐α in both NMRI and L66^+/−^ mice, both singly and when combined with memantine, compared to the matching vehicle‐treated controls (Fig. 6A). Neither memantine nor 5 mg·kg^−1^ HMTM (alone or in combination) altered the levels of TNF‐α in either genotype relative to controls (Fig. 6A).

There were no treatment differences in the levels of IL‐10 in L66^+/−^ mice (Fig. 6B). However, IL‐10 in vehicle‐treated L66^+/−^ mice was already significantly elevated in comparison to NMRI mice. In NMRI mice, both memantine and HMTM (5 and 15 mg·kg^−1^) increased levels of IL‐10 (Fig. 6B). This sustained increase in IL‐10 was unaffected by combination treatment. These data suggest that HMTM produced a dose‐dependent disruption of the balance between counteracting cytokines ameliorating the inflammatory status of the brain. This can be seen as a significant inverse correlation between the levels of TNF‐α and IL‐10 (*r* = −0.218, *P* = 0.028; Fig. 6C), even after a long washout period.

### The levels of truncated tau fragments correlate with pro‐inflammatory cytokine status of the brain

**Fig. 7:**
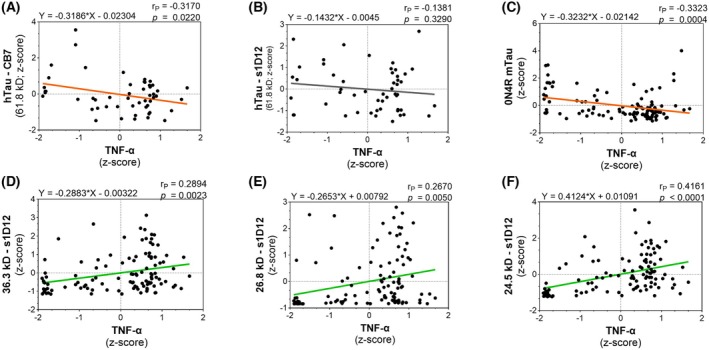
Truncated tau fragments correlate with increased TNF‐α. Correlations between the levels of TNF‐α and FL‐hTau (CB7) (A), FL‐hTau (s1D12) (B), FL‐mTau (C), 36.3 kD tau fragment (D), 26.8 kD tau fragments (E) and 24.5 kD tau fragments (F). TNF‐α levels were inversely correlated with N‐terminal human tau and murine tau but positively correlated with the three truncated tau fragments (24.5, 26.8 and 36.3 kD). All values (A, B – hTau/L66^+/−^: *n* = 52; C–F – mTau and truncated tau: *n* = 109) were transformed into *z*‐scores and differences were considered statistically significant when *P* < 0.05.

To explore whether the effects of the drug administration on the levels of TNF‐α could be attributed to changes in tau pathology, a whole cohort analysis was performed on the correlation of the levels of FL‐tau (murine and human) and truncated tau fragments, with the levels of the pro‐inflammatory cytokine (Fig. 7). TNF‐α was inversely correlated with the N‐terminal hTau and mTau (Fig. 7A,C), but not with repeat‐domain FL‐hTau (Fig. 7B). However, TNF‐α was found to be positively correlated with all three of the truncated tau fragments that were quantified (24.5, 26.8 and 36.3 kD; Fig. 7D–F). This strongly suggests that the truncated tau fragments may be driving neuroinflammatory responses that, in turn, can be counteracted by HMTM (15 mg·kg^−1^; Fig. 6A) either singly or as an add‐on to memantine administration.

## Discussion

In the present study, we have investigated the impact of HMTM treatment on inflammatory markers in a mouse model of FTD following a lengthy drug washout period. This is the first study investigating the long‐term effects of HMTM following cessation of dosing. Since it was previously reported that symptomatic therapies for dementia interfered with the disease‐slowing effects of HMTM in AD [30], we at the same time investigated whether memantine could sustain any long‐term effects in brain inflammation and/or negatively impact on the effects of HMTM following the halting of administration of both drugs. Understanding the long‐term effects of HMTM, both alone and combined with memantine, is all the more relevant in light of recent reports describing the worsening of biomarkers and cognition after discontinuation of long‐term treatment of patients with amyloid‐based treatments with lecanemab and donanemab [48]. We were primarily interested in evaluating changes in the levels of pathological tau species following a period of drug washout, as well as investigating secondary pathological mechanisms relevant to tissue homeostasis, such as glial function and brain inflammation, that are known players in several forms of dementia. We found that the levels of truncated tau species remained lower in mouse brains 12 weeks after the last administration of HMTM. The long‐lasting lowering effect of HMTM on the levels of truncated tau was neither replicated nor prevented by memantine when administered singly or in combination respectively. Additionally, we report a sustained, dose‐ and tau‐dependent lowering of the pro‐inflammatory cytokine TNF‐α, accompanied by tau‐independent microglial activation following HMTM treatment. While the results suggest a dual mechanism of action for HMTM in glial cell response and the levels of cytokines, memantine also demonstrated different interactions with the long‐term effects of HMTM. Memantine prevented the HMTM‐mediated microglial cell reactivity but did not interfere with the inflammation‐lowering effect of HMTM. This difference might be accounted for by the difference in tau dependence between glial cell reactivity and the levels of inflammatory cytokines; memantine did not impact the reduction in the levels of truncated tau fragments caused by HMTM. By enhancing microglial reactivity, HMTM may favour the clearance of abnormal tau aggregates in an environment where inflammation is reduced.

### HMTM‐induced long‐lasting decrease in truncated tau

It has been reported previously that there is a reduction in pharmacological activity of HMTM when given as an add‐on to rivastigmine, a symptomatic treatment for AD [33]. Treatment with HMTM increases basal levels of acetylcholine levels in the hippocampus in wild‐type mice, facilitates metabolic flow of substrates and enhances mitochondrial electron transport, with each of these effects being prevented to some extent by symptomatic drugs for dementia [32, 49]. HMTM has a normalising effect on the expression levels of synaptic proteins of the SNARE complex, an effect that is diminished in combination with rivastigmine [50]. The negative interaction of these drugs either had no effect or only slightly attenuated the HMTM‐triggered decrease in tau pathology [33]. These studies provide a neuropharmacological explanation for the unexpected poor clinical response to HMTM when given as an add‐on to symptomatic treatments [30]. It suggests that the chronic brain stimulation produced by symptomatic drugs results in a compensatory homeostatic downregulation in multiple neuronal systems, leading to a reduction in the pharmacological efficacy of HMTM in the brain [32, 33, 49]. We here demonstrate that measurement of FL‐tau may not always be sufficient to evaluate the efficacy of putative tau‐lowering medications. We identified three truncated tau fragments spanning from the proline‐rich domain to the C‐terminus of the protein, which provide more sensitive indicators of target engagement, especially when normalised to FL‐tau levels. One of these fragments, the 36.3 kD tau band, was the only tau fragment showing treatment effects solely in L66^+/−^ mice. This could potentially offer a biomarker for monitoring treatment. Importantly, we also extend our understanding of the mechanism of action of HMTM. In contrast to what happens in treatment, where HMTM‐induced decrease in tau pathology is attenuated by symptomatic treatments [51], no long‐lasting negative interaction in terms of tau pathology was observed after drug washout. Furthermore, HMTM sustained a long‐term decrease in the levels of truncated tau fragments in the brain. Therefore, it was important to understand if other tissue homeostatic mechanisms, secondary to tau pathology, such as brain inflammation, could remain altered after a period of drug washout, and whether any negative interactions had long‐term consequences.

There are conflicting reports about the role of microglia in neurodegenerative disease. It has been reported that microglial activation has detrimental effects on the brain by inducing toxic, chronic neuroinflammation [52]. In contrast, microglial activation is crucial for clearance of pathological tau aggregates since microglia can internalise and degrade abnormal aggregates, promoting neuronal survival. A study of individuals with underlying AD pathology in the absence of clinical dementia reported that microglial activation protected against accumulation of tau aggregates [53], highlighting a key role for microglia in maintaining brain health. In tauopathies, filamentous tau inclusions are mainly found in neurons but they are also found in glial cells in chronic traumatic encephalopathy, progressive supranuclear palsy, corticobasal degeneration and globular glial tauopathies [54].

Neuroinflammatory changes have been reported in PS19 mice in which tau with the FTD‐associated P301S mutation is expressed in both neuronal and glial cells [23]. Here, however, we have used L66^+/−^ mice in which P301S tau expression is restricted to neurons [38] and have observed similar levels of microglia (Iba1) and astrocytes (GFAP) reactivity, and of the pro‐inflammatory cytokine TNF‐α in L66^+/−^ and wild‐type mice. We have previously reported glial activation and neuroinflammatory profiles in homozygous L66^+/+^ mice with activation of microglia occurring in most regions of the basal forebrain and the hippocampus, and this change was more prominent than astroglial activation [55]. In the present study, we have used heterozygous L66^+/−^ mice of a similar age, in which the mice have a lesser tau burden than their homozygous counterparts [38] and likely exhibit less pronounced glial cell activation and neuroinflammatory profiles. Regional‐ and cell‐specific changes in mouse brains may not be readily quantifiable if they are not generally widespread in the tissue. A recent study reported that microglia showed accelerated ageing across brain regions [56]. Nevertheless, the ageing rate, measured as the transcriptional signature of 82 age‐related selected genes, was reported to be greater in the cerebellum and striatum than in hippocampus and cortex, leading to the conclusion that transcriptional ageing of microglia is regionally dependent [56]. Taken together, these findings would explain the lack of measurable microglial phenotypic differences between L66^+/−^ and wild‐type mice using biochemical methods of analysis that do not allow for the determination of region‐specific changes. Furthermore, we show that the levels of the immunosuppressive cytokine IL‐10 are elevated in the brain of L66^+/−^ mice, which likely is part of a protective response to the accumulation of aggregated tau in neurons. IL‐10 deficiency can potentiate tau pathology in response to acute systemic inflammation, suggesting a primary protective role for IL‐10 in preventing tau pathology in the brain [57]. The absence of a substantial neuroinflammatory response in L66^+/−^ mice is likely due to elevated levels of neuroprotective IL‐10 in L66^+/−^ when compared with wild‐type mice. Some drug effects may be neither genotype nor tau pathology dependent. A more extensive inflammatory profiling of the L66^+/−^ mice would be necessary to fully establish the complexity of the brain immune response to the overexpression of the human mutant tau.

### Memantine prevents a sustained HMTM tau‐independent microglial activation

GFAP levels did not change in response to HMTM or memantine, either singly or combined, suggesting that astrocytic reactivity remained unaltered. The levels of Iba1 in both NMRI and L66^+/−^ mice, however, were increased by both drugs, except when administered together. Despite the effect of the treatments in L66^+/−^ mice, our correlation analysis suggests that the effects were tau independent. Therefore, it is less surprising that, while symptomatic treatments did not interfere with the HMTM decrease in tau pathology, there was a pharmacological interference by memantine on the sustained HMTM‐induced microglial activation which is not solely a response to variations in the levels of tau. Despite the tau‐independent effect of HMTM in modulating microglial activation, we cannot ignore the intricate interplay between tau pathology and neuroinflammation, considering that effects may differ between disease stages during ongoing treatment. Importantly, the nature of the pharmacological interference may also vary depending on whether it results from direct action of HMTM and symptomatic treatments on specific cellular processes or a response to amelioration of tau pathology. Our evidence for interference of symptomatic treatments with HMTM‐mediated effects suggests that the tau aggregation inhibition of HMTM is less susceptible to interference than other secondary mechanisms.

### HMTM ameliorates brain inflammation in a tau‐dependent manner

TNF‐α is a key inflammatory cytokine, playing a role in a multitude of pathogenic processes, ranging from external infections by foreign agents, to diseases caused by the dysregulation of intrinsic cellular mechanisms, such as in neurodegenerative diseases [58]. The production of TNF‐α in response to acute processes acts as a protective mechanism, but if there is no resolution of inflammation, and the levels of TNF‐α become chronically elevated, it becomes deleterious, preventing tissue repair and regeneration [59]. In contrast, IL‐10 is an anti‐inflammatory cytokine, during the immune‐resolution stage, counteracting chronic inflammation processes and tissue damage [60]. Our data suggest that, following cessation of HMTM treatment, there is a shift in immunomodulation towards a neuroprotective response. HMTM decreases the levels of TNF‐α and produces a switch towards an anti‐inflammatory response with increased IL‐10. The HMTM‐induced increase in IL‐10 was only observed in NMRI mice, since basal levels of the immunosuppressive cytokine were already elevated in L66^+/−^ mice, likely in response to the expression of mutated tau transgene. Therefore, whereas the effect of HMTM in increasing IL‐10 seems to be tau independent, we cannot discard the possibility that HMTM may also be able to promote an increase in immunosuppressive interleukins by a tau‐dependent mechanism in a more sustained manner after ceasing drug administration, thus preventing chronic neuroinflammation. In support of this is the fact that HMTM maintained decreased levels of TNF‐α in both NMRI and L66^+/−^ mice, while the levels of TNF‐α were significantly correlated with truncated levels of tau and correlated inversely with the levels of IL‐10. MTC has previously been reported to suppress neuroinflammation and improve neurological function in an animal model of stroke, upregulating IL‐10 and downregulating TNF‐α expression [40]. HMT diffuses passively across membranes, along a concentration gradient, reaching the blood, where it can be taken up by red blood cells, thereby bypassing first‐pass liver metabolism [25]. This differs from the delivery of MTC, also referred to as methylene blue, in that this provides the oxidised form MT^+^, which is absorbed less and metabolised to a greater extent, hence having poorer pharmacokinetic properties [25]. The greater bioavailability of HMT in blood and brain likely results in enhanced modulation of inflammatory pathways.

In summary, HMT triggers a dose‐dependent prolonged increase in microglial reactivity, in a context of lowered cytokine inflammatory profile, that continues after cessation of treatment and these changes may be important in the clearance of abnormal protein aggregates and supporting tissue repair. Our findings demonstrate that the tau‐independent effects of HMT on microglial response are prevented by pre‐treatment with memantine and illustrate that pharmacological interference of the central immune response can still have an effect even after cessation of treatment.

## Materials and methods

### Chemicals, antibodies and assay kits

All reagents used, unless otherwise stated, were obtained from Sigma‐Aldrich, Dorset, UK, and were of the highest grade of purity commercially available. Memantine hydrochloride, batch N.10, was acquired from Tocris Bioscience, Bristol, UK. HMTM (batch 9751SR114; 99.4% pure) was supplied by TauRx Therapeutics Ltd., Aberdeen, UK. The anti‐tau antibodies CB7 and s1D12, recognising epitopes within the N‐terminus (13–25) and the core repeat domain (341–353) of tau, respectively, were produced by the Scottish Biologics Facility (Aberdeen, UK) [58, 61]. Antibody anti‐GFAP (Cat. #60190‐1‐lg) was obtained from Proteintech Europe (Manchester, UK). Secondary antibodies anti‐mouse IgG HRP conjugated (Cat. #1706516) and anti‐rabbit IgG HRP conjugated (Cat. #1721019) were both obtained from Bio‐Rad Laboratories Ltd. (Watford, Hertfordshire, UK). The murine tumour necrosis factor alpha (TNF‐α) Standard ABTS ELISA kit (Cat. #900‐K54) and murine interleukin‐10 (IL‐10) Mini ABTS ELISA Development kit (Cat. #900‐M53) were obtained from Peprotech EC, Ltd. (London, UK). The mouse AIF/Iba1 sandwich ELISA kit (Cat. #LS‐ F52885) was purchased from LSBio (Shirley, MA, USA). Recombinant hT441 (Cat. #T‐1001‐2, Lot. #R071919T441) was obtained from rPeptide (Watkinsville, GA, USA).

### Expression and purification of the tau fragment 186–391

Tau (186–391) was expressed in *Escherichia coli* using a pRK172 plasmid in which the cDNA sequence, with an added N‐terminal Met start codon, was subcloned from pRK172‐htau40 and DNA sequence confirmed. The protein was purified using the same method used to purify tau (297–391) [62].

### Animal model

Naval Medical Research Institute (NMRI)‐derived, heterozygous L66^+/−^ transgenic mice were used alongside their respective background, NMRI wild‐type control (may be referred to as NMRI or WT mice). The L66 mouse line expresses FL hTau (2N4R isoform; 441 amino acids) carrying P301S and G335D mutations and expression of tau is driven by the neuronal‐specific Thy‐1 promotor [38]. These mice show early‐onset and high tau load throughout the brain, and their genetic, behavioural and histopathological phenotypes are reminiscent of features associated with FTD [38].

Female transgenic heterozygous L66^+/−^ (*n* = 60) and wild‐type NMRI (*n* = 60) litters were supplied by Charles River (Margate, UK). Following transportation to the Medical Research Facility, University of Aberdeen, by road about 1 month prior to use to allow for ample acclimatisation, mice were colony housed (up to 5 per cage) in wire‐lid cages (Tecniplast S.p.A, Buguggiate, Italy) with corn cob bedding and enrichment (paper strips and cardboard tubes: DBM Scotland Ltd., Broxburn, UK) in a controlled facility. Mice were housed in treatment groups and exposed to a 12‐h light/dark cycle with lights turned on at 7:00 AM and off at 7:00 PM. Standard housing conditions were maintained (temperature = 21 ± 1 °C and 50–65% relative humidity) with food pellets and water provided *ad libitum*. Experiments were performed in accordance with the European Community Council Directive (63/2010/EU), and a project licence (number: 7008396) was reviewed by the University of Aberdeen Animal Welfare and Ethical Review Body (AWERB) and approved by the Home Office on 30 January 2015. All the approved procedures were in accordance with the University Code of Practice for Research Involving the Use of Animals, compliant with the ARRIVE guidelines, and under the UK Animals (Scientific Procedures) Act (1986).

### Experimental design

**Fig. 8:**
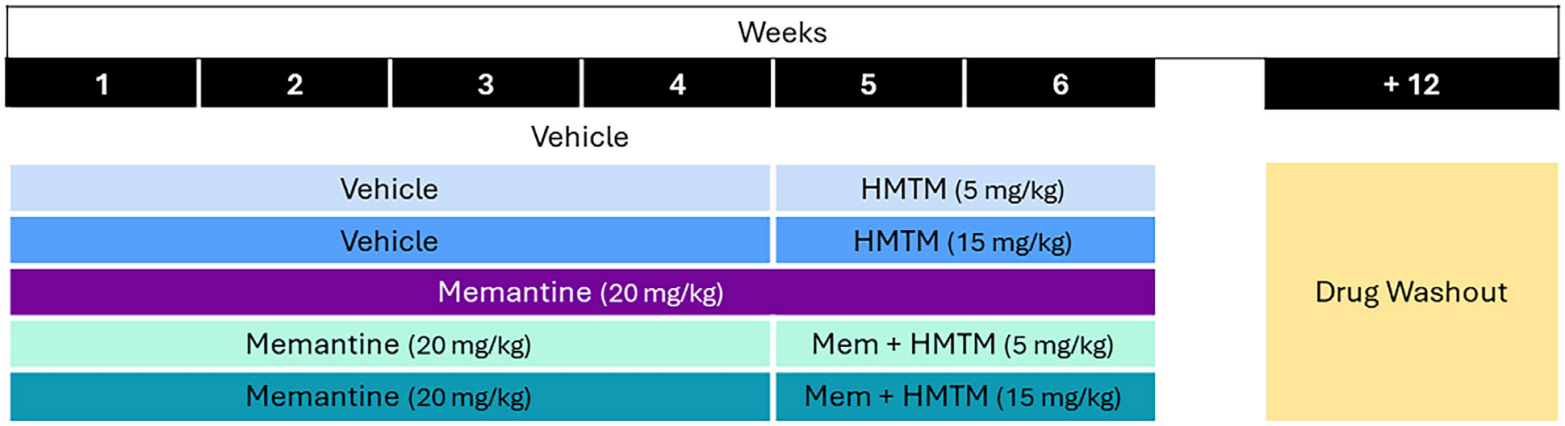
Study design and administration regime for drug treatments. Saline or memantine (20 mg·kg^−1^) was given to female mice aged 5–6 months for the first 4 weeks. Following this, treatment continued with or without HMTM (5 or 15 mg·kg^−1^) for an additional 2 weeks. Following a 12‐week washout period after cessation of treatment, tissue was harvested. All treatments were given by oral gavage, 5 days per week.

At the beginning of the protocol, all mice were 5–6 months old. The different groups and therapy regimes are displayed in Fig. 8. All treatment regimens were administered to mice by oral gavage, 5 days per week (Mon–Fri), over a 6‐week period. Either water or memantine (20 mg·kg^−1^) was given daily by oral gavage for the first 4 weeks. Following this, treatment continued with or without HMTM (5 or 15 mg·kg^−1^) for an additional 2 weeks. In weeks 4 and 6, a brief behavioural test (measurement of startle response amplitude) was performed (data not shown). Brain tissue was collected 12 weeks after the last injection (washout period) and snap frozen in liquid nitrogen. All tissues were kept at −80 °C until further use. The absence of the active MT moiety in the tissue was confirmed using HPLC with LC–MS/MS detection as described elsewhere (data not shown) [49]. Following the start of the study (*n* = 120), five mice had to be excluded from the study due to weight reductions of 20%, in accordance with Home Office regulatory requirements. A total of 115 mice reached the end of the drug administration period, and 110 were then considered for data analysis (*n* = 7–10 per experimental group, see Table 2 for details).

**Table 2:** Summary of the total number of mice (*n*) tested for each experimental group. HMTM, hydromethylthionine mesylate.

Genotype	Treatment (mg·kg^−1^)		Group size (*n*)
	Memantine	HMTM	
NMRI	0	0	10
	20	0	9^a^
	0	5	10
	0	15	10
	20	5	8^a^, ^b^
	20	15	10
L66^+/−^	0	0	9^a^
	20	0	10
	0	5	9^b^
	0	15	9^a^
	20	5	9^b^
	20	15	7^b^, ^c^

^a^ One mouse was not used for data analysis due to wrong assignment of the genotype to the respective experimental group.

^b^ One mouse excluded during the *in vivo* stage due to body weight loss of at least 20% of the initial body weight (regulatory compliance).

^c^ One sample was missing.

### Tissue homogenisation

The cerebellum was removed from the left hemisphere of brain, and brain tissue was homogenised in RIPA buffer 1× (Cell Signalling Technology Europe B.V., Cat. #9806, Leiden, The Netherlands), supplemented with PMSF (1 mm; Cell Signalling Technology Europe B.V., Cat. #8553) and with protease and phosphatase inhibitor mini tablets (Thermo Fisher Scientific, Loughborough, UK, Cat. #A32959). The homogenate was then transferred to microcentrifuge tubes and mixed by rotation for 90 min at 4 °C. Each sample then underwent three snap‐freeze/thaw cycles in liquid nitrogen before being centrifuged (10 000 ***g***, 4 °C, 10 min) and the supernatant fraction was stored at −80 °C until further use.

### SDS/PAGE and immunoblotting analysis

Protein was determined using the BCA Protein Assay Kit (Thermo Fisher Scientific, Cat. #23225) as per manufacturer's instructions. Samples were measured in triplicate, in sterile, 96‐well plates using a standard four‐parameter logistic curve obtained from the use of incremental known concentrations of bovine serum albumin (2–22 μg). SoftMaxPro7 software (SpectraMax 190, Molecular Devices, Wokingham, Berkshire, UK) was used to determine protein concentrations in samples.

Samples were prepared for SDS/PAGE with 1× reducing sample buffer from a 5× stock (Thermo Fisher Scientific, #39000 – supplemented with 100 mm DTT) and treated for 5 min at 95 °C. Samples (20 μg protein per lane) were applied and separated by electrophoresis (120 V for 20 min followed by 180 V for 30 min) using 18‐ or 26‐well, 8–16% pre‐cast TGX midi gels (Bio‐Rad Lab. Ltd., Cat. #5671104) and Tris‐glycine running buffer (25 mm Tris, 200 mm glycine and 0.1% SDS).

Proteins were then transferred to PVDF membranes (Bio‐Rad Lab. Ltd., Cat. #1704272) for GFAP detection, and to nitrocellulose membranes (Bio‐Rad Lab. Ltd., Cat. #1704271) for tau detection, using the Trans‐Blot Turbo Transfer System (Bio‐Rad Lab. Ltd., Cat. #1702150) and Trans‐Blot Turbo Transfer buffer (Bio‐Rad Lab. Ltd., Cat. #10026938) according to the manufacturer's instructions. Membranes were blocked for 1 h at room temperature (RT) in 5% dried skimmed milk powder in TBS‐T (50 mm Tris, 150 mm NaCl, pH8 and 0.1% Tween 20). Membranes were incubated overnight at 4 °C with primary antibodies against GFAP (1 : 5000) and the antibodies CB7 (1 : 1000) and s1D12 (1 : 10 000) against tau. Following overnight incubation with primary antibody, membranes were washed three times for 15 min in TBS‐T and then incubated with secondary antibody (anti‐mouse IgG; 1 : 5000 for detection of GFAP and 1 : 10 000 for detection of tau), in addition to Strep‐Tactin HRP conjugate (Bio‐Rad Lab. Ltd., Cat. #1610380) for detection of Precision Plus Protein Western C standards (Bio‐Rad Lab. Ltd., Cat. #161‐0385) diluted 1 : 10 000 for 2 h at RT. Signal intensities for all protein bands of interest were analysed for each membrane using uvp vision works ls 8.2 (Life Sciences Software from UVP; Analytik Jena US LLC, Upland, CA, USA) and normalised with respect to the signal intensity of total protein per lane.

Total protein staining for GFAP membranes was performed using the MemCode™ Reversible Protein Stain kit – for PVDF membranes (Thermo Fisher Scientific, Cat. #24585; performed following manufacturer's recommended procedures).

Total protein for tau membranes was detected following the reaction between the trihalo compound present in the Any kD™ Stain‐Free™ pre‐cast TGX midi gels and the tryptophan residues present in the proteins to form fluorescent tryptophan adducts, triggered by exposure to UV light for 1 min. The total protein staining images were obtained after transfer of the protein to the nitrocellulose membranes. As reference for intermembrane variability and absolute determination of tau quantities in the samples, recombinant hTau441 (rPeptide) was reconstituted in water to 1 mg·mL^−1^ and a series of dilutions prepared to load into the gel 2–100 ng of tau. A four‐parameter logistic regression curve was fitted and used to estimate the absolute quantities of tau in the samples using the tau antibodies CB7 (Fig. S1A) and s1D12 (Fig. S1B). It is important to note that the best estimate of the quantity of tau in the mice samples is for the FL hTau, however, the same curve was used to extrapolate the quantity of mTau and tau fragments detected using s1D12.

### Immunoprecipitation and mass spectrometry

Tau was immunopurified from the total soluble protein extracts from brains of vehicle‐dosed NMRI and L66^+/−^ mice, prior to in‐gel trypsin digestion of the proteins for identification and database match of the resulting peptides. Recombinant hTau441 and Tau186‐391 were subjected to the same workflow and processed as controls for database‐match identification. The efficacy of the purification by immunoprecipitation was confirmed by immunoblotting the immunoprecipitate (IP) and flow‐through (FT) fractions using both s1D12 (Fig. S2A) and s1G2 (Fig. S2B) antibodies and total protein staining by Sypro® Ruby (Thermo Fisher Scientific; Cat. #S12000) (Fig. S3). Two NMRI and two L66^+/−^ samples were used and ion scores for each band from both samples were averaged to produce a heatmap for sequence coverage. The workflow is detailed as follows.

#### Immunoprecipitation

Immunoprecipitation was carried out using a Pierce Crosslink Magnetic IP/Co‐IP kit (Thermo Fisher Scientific, Cat. #88805) to covalently conjugate and crosslink the s1D12 to A/G magnetic beads, following the manufacturer's instructions. S1D12 (5 μg) was added to magnetic beads (25 μL) and incubated for 15 min at RT. The beads were then collected and supernatant was discarded. The beads were then washed (3×) with the supplied coupling buffer, re‐suspended in coupling buffer and then incubated with DSS (20 μm) for 30 min at RT. After collecting the beads, elution buffer was added to remove/wash the non‐crosslinked antibody, repeating this step 3× and finally suspending the beads in IP lysis/wash buffer. Total protein (200 μg) from brain extracts and 20 μg of the recombinant peptides dGAE, Tau186‐391 and hTau441 were incubated for 1 h at RT in a rotator. The bead conjugates were then washed with IP lysis/wash buffer (2×) and water (1×), followed by elution to release the antigen and collection of beads. Lastly, elution buffer was neutralised with neutralisation buffer (10 μL per 100 μL of sample). The IP was separated using SDS/PAGE, and the polyacrylamide gel was stained using Sypro® Ruby following the suggested manufacturer's procedure without modification. Gel bands were excised and subjected to in‐gel tryptic digestion.

#### In‐gel protein digestion

Gel slices were chopped into 1 mm cubes and placed into polypropylene 96‐well, V‐bottom, low‐ binding sample processing microplates (Thermo Fisher Scientific, Cat. #249944). An automated in‐gel digestion protocol was carried out using a JANUS liquid handling workstation (PerkinElmer) and a custom program based on the method of Shevchenko *et al*. [63]. The enzyme was trypsin (Promega sequencing grade V5111; Chilworth Southampton, Hampshire, UK). Peptide solutions that had been eluted from the gel pieces were pooled if the original gel slices were split between multiple wells, then frozen at −70 °C, dried by vacuum centrifugation (SpeedVac) and resuspended with 10 μL 0.1% trifluoroacetic acid.

#### LC–MS

The LC–MS system comprised an UltiMate 3000 RSLCnano coupled to a Q Exactive Plus (Thermo Fisher Scientific). Peptides (8 μL) were injected via a sample loop using loading pump buffer A (0.1% TFA in ultrapure water) at 10 μL·min^−1^ and bound to the C18 PepMap 100 precolumn (300 μm × 5 mm, Thermo Fisher Scientific,Cat. #160454). After 5 min, flow was switched to the nano pump and peptides were reverse flushed to the PepMap RSLC C18 column (75 μm × 25 cm, Thermo Fisher Scientific, Cat. #ES801) and separated with a gradient of acetonitrile increasing from 2.4% to 32% in 35 min. Nano pump solvent A was 0.1% formic acid in ultrapure water while nano pump solvent B was 0.1% formic acid in 80% acetonitrile. The column was washed for 8 min in 90% solvent B and then equilibrated for 15 min in 3% solvent B prior to the next injection.

The Q Exactive Plus was operated in positive polarity mode using a Top 10 data‐dependent acquisition (DDA) method. Full MS scan parameters were as follows: resolution = 70 000; AGC target = 3e6; maximum IT = 50 ms; scan range = 375–1750 *m*/*z*. dd‐MS^2^ parameters were as follows: resolution = 17 500; AGC target = 5e4; maximum IT = 100 ms; loop count = 10; isolation window = 1.6 *m*/*z*; NCE = 28; charge exclusion ≤ 2 and > 5; peptide match = preferred; and dynamic exclusion = 40 s.

#### Peptide‐match data analysis

MS raw data files, acquired between 5 and 65 min after flow switching, were processed using Proteome Discoverer (version 2.2, Thermo Fisher Scientific) and a workflow that incorporated Mascot Server (version 2.6, Matrix Science, London, UK), target decoy peptide‐spectrum match validation (strict false discovery rate of 0.01) and peptide quantification by precursor ion peak area. The search settings were ESI‐FTICR as instrument and the database consisted of 37 525 human and mouse protein sequences downloaded from UniProtKB (access date 30/11/2022) and including 8 hTau isoforms (fetal, isoforms A‐G) and 5 mouse tau isoforms (isoforms A‐E). Trypsin was entered as the enzyme and two missed cleavage sites were allowed, carbamidomethyl (C) was set as fixed modification and oxidation (M) as variable modification. The peptide tolerance was set to 10 ppm, minimum peptide length to 4 and level of confidence to at least ‘high’.

### Determination of TNF‐α

Murine TNF‐α Standard ABTS ELISA Development kit was used to detect TNF‐α levels in samples according to manufacturer's instructions. Briefly, capture antibody (1 μg·mL^−1^ in PBS) was added to each ELISA plate well and incubated overnight at RT. Wells were aspirated and washed four times with wash buffer (0.05% Tween‐20 in PBS). Blocking buffer (1% BSA in PBS) was then added to each well and plate was incubated for 1‐h at RT. The plate was aspirated and washed four times. Murine TNF‐α standard (1 μg·mL^−1^) was serially diluted (2000–62.5 pg·mL^−1^ in 0.05% Tween‐20 and 0.1% BSA in PBS) to create a standard curve. Each standard and sample were included in triplicate on the plate and incubated at RT for 2 h. The plate was aspirated and washed four times. The detection antibody (0.5 μg·mL^−1^ in 0.05% Tween‐20 and 0.1% BSA in PBS) was added to each well and the plate was incubated for 2 h at RT. The plate was aspirated and washed four times. Avidin‐HRP conjugate was added to wells and incubated for 30 min at RT. The plate was aspirated and washed four times. Lastly, the ABTS substrate solution was added to each well and the plate was monitored at RT for colour development using an ELISA plate reader (Spectramax 190, Molecular Devices) at 405 nm with wavelength correction set to 650 nm. The concentration of TNF‐α in the samples was determined from the TNF‐α standard serial dilution by using a four‐parameter logistic equation.

### Determination of IL‐10

Murine IL‐10 Mini ABTS ELISA Development Kit was used to detect IL‐10 levels in samples according to manufacturer's instructions. Briefly, capture antibody (2 μg·mL^−1^ in PBS) was added to each ELISA plate well and incubated overnight at RT. Wells were aspirated and washed four times with wash buffer (0.05% Tween‐20 in PBS). Blocking buffer (1% BSA in PBS) was then added to each well and plate incubated for 1 h at RT. The plate was aspirated and washed four times. Murine IL‐10 standard (1 μg·mL^−1^) was serially diluted (5000–39.1 pg·mL^−1^ in 0.05% Tween‐20 and 0.1% BSA in PBS) to create standard curve. Each standard and sample were included in triplicate on the plate and incubated at RT for 2 h. The plate was aspirated and washed four times. The detection antibody (0.5 μg·mL^−1^ in 0.05% Tween‐20 and 0.1% BSA in PBS) was added to each well and plate incubated for 2 h at RT. The plate was aspirated and washed four times. Avidin‐HRP conjugate was added to wells and incubated for 30 min at RT. The plate was aspirated and washed four times. Lastly, the ABTS substrate solution was added to each well and the plate was monitored at RT for colour development with ELISA plate reader (Spectramax 190, Molecular Devices) at 405 nm with wavelength correction set to 650 nm. The concentration of IL‐10 in the samples was determined from the IL‐10 standard serial dilution by using a four‐parameter logistic equation.

### Determination of Iba1

The mouse AIF/Iba1 sandwich ELISA kit was used to detect the levels of Iba1 in the study as per manufacturer's instructions. Briefly, the samples were loaded into a pre‐coated 96‐well plate with the capture antibody specific for capturing mouse Iba1. The plate was incubated for 2 h at 37 °C. After the incubation period, the well content was aspirated and the wells were loaded with the biotinylated detection working solution, followed by a 1 h incubation at 37 °C. The plate was then washed three times, letting the plate sit for 2 min between each wash, followed by aspiration of the washing buffer. For the following step, the HRP‐avidin conjugate was loaded onto the wells and incubated for 1 h at 37 °C. The plate was once again washed five times for 2 min each wash, and lastly developed with 3,3′,5,5′‐tetramethylbenzidine substrate for 15 min at 37 °C, protected from light, followed by the addition of the STOP solution, changing the colour from blue to yellow. The absorbance was read at 450 nm using a correction wavelength set at 540–570 nm. The optical density reading values were introduced in a four‐parameter logistic regression equation determined from a standard curve using the Iba1 standard supplied with the kit to obtain the amount of Iba1 in the samples. Values were normalised per total amount of protein used in the assay.

### Statistical analysis

Statistical analyses were undertaken using graphpad prism (v. 10.0.3; GraphPad Software Inc., San Diego, CA, USA). Outlier detection was performed (ROUT, Q = 1%), followed by testing of the normality of distribution of the data, using the Shapiro–Wilk test. Where the data followed a non‐normal distribution, the median and interquartile range (IQR) were represented in box‐and‐whiskers plots and the differences between groups were determined by the Kruskal–Wallis test, followed by a *post hoc* Dunn's test for multiple comparisons. Otherwise, the data are shown as bar graphs and differences tested by one‐way ANOVA, followed by *post hoc* Tukey test for multiple comparisons. To establish correlation between FL tau and tau fragments, where both variables were found to have a normal distribution, a Pearson correlation and simple linear regression were applied. If the data were not Gaussian, a non‐linear regression was applied, and the best‐fit model was determined by Akaike's information criterion (AICc). Following this, the variables were logarithmically transformed accordingly. A Pearson correlation and simple linear regression were then performed. To test for existence of differences between the ratios of fragmented to FL tau, a grouped two‐way ANOVA was used, followed by a *post hoc* Tukey test for multiple comparisons. For the correlation analysis between the levels of tau and inflammatory markers, the data were first transformed to *z*‐scores and then the Pearson coefficient of correlation was determined. The linear relation between the variables was also explored. *P*‐values less than 0.05 were considered statistically significant.

## Conflict of interest

The sponsor was involved in the design of the study; the collection, analysis and interpretation of the data; and the writing of the report. The corresponding author had full access to all the data and had final responsibility for the submission of the report for publication. CMW and CRH are employed officers in TauRx Therapeutics Ltd., an affiliate of WisTa Laboratories Ltd. CH is employed by TauRx Therapeutics Ltd. GR has received funding from TauRx Therapeutics Ltd. RL, VM, GR, CMW and CRH are named inventors on patents and patent applications owned by WisTa Laboratories Ltd.

## Author contributions

RXS, LR, GR and CRH conceived and designed the study. VM, LR, ED, PA and GR managed the *in vivo* stage of the study, administered the drugs and harvested the tissue. RXS, SHL, RL, ML, TV and EAG were responsible for data acquisition and analysis. RXS, GR, CMW and CRH interpreted the data. RXS and CH prepared the initial draft of the manuscript. RXS, CH, GR, CMW and CRH prepared, scientifically revised and edited the manuscript. GR, CMW and CRH were responsible for funding acquisition. All authors reviewed and approved the final version of the manuscript.

### Peer review

The peer review history for this article is available at https://www.webofscience.com/api/gateway/wos/peer‐review/10.1111/febs.70021.

## Supporting information


**Fig. S1.** Four‐parameter logistic regression curves used to estimate the absolute quantities of tau in samples.
**Fig. S2.** Verification of the efficacy of the immunoprecipitated tau samples further processed by MS/MS.
**Fig. S3.** Total protein staining by SYPRO Ruby gel of the immunoprecipitated fractions in L66^+/−^ and NMRI mice.

**Table S1.** Raw mass spectrometry data.

## Data Availability

The data that support the findings of this study are available from the corresponding authors upon reasonable request.
