# Supplementary material for: Hydromethylthionine sustains truncated tau‐dependent inflammation‐lowering effects in mouse brain
Source: FEBS J. 2025 Feb 17;292(10):2602–23. doi: 10.1111/febs.70021 (PMC12103070; doi:10.1111/febs.70021)
Supplement: Supplementary file 1 — Fig. S1. Four‐parameter logistic regression curves used to estimate the absolute quantities of tau in samples. Fig. S2. Verification of the efficacy of the immunoprecipitated tau samples further processed by MS/MS. Fig. S3. Total protein staining by SYPRO Ruby gel of the immunoprecipitated fractions in L66+/− and NMRI mice. [file FEBS-292-2602-s002.pdf]

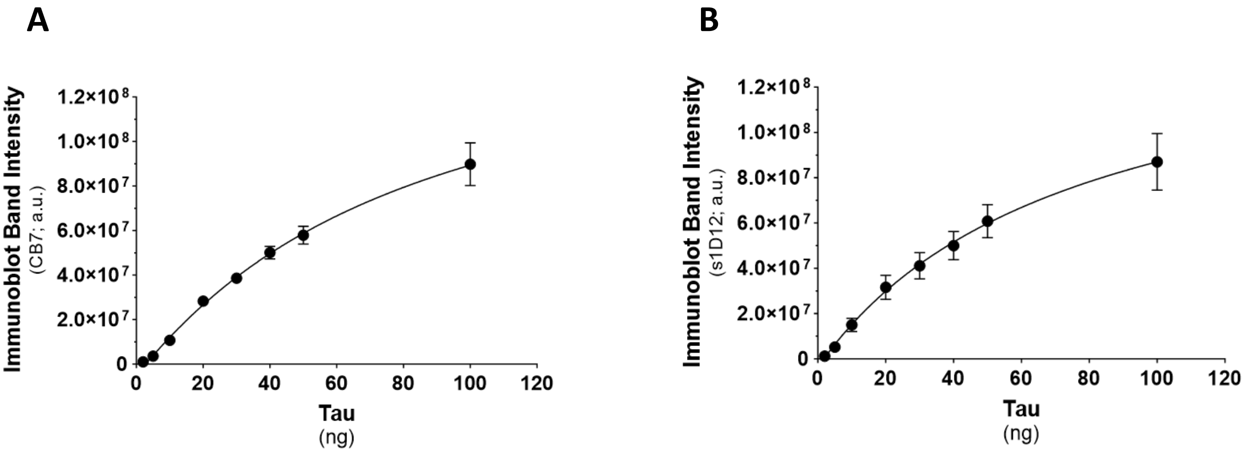

$$y = -4.29 \times 10^{6} + \frac{1.68 \times 10^{8}}{1 + \left(\frac{x}{80.89}\right)^{1.07}}$$

$$y = -3.72 \times 10^{6} + \frac{1.94 \times 10^{7}}{1 + \left(\frac{x}{76.44}\right)^{0.99}}$$

Figure S1: 4-parameter logistic regression curves used to estimate the absolute quantities of tau in samples. Standard curves for CB7 **(A)** and s1D12 **(B)** antibodies. Relevant equations displayed below plots. Data are expressed as mean ± SEM values for all the blot membranes labelled with either CB7 or s1D12.

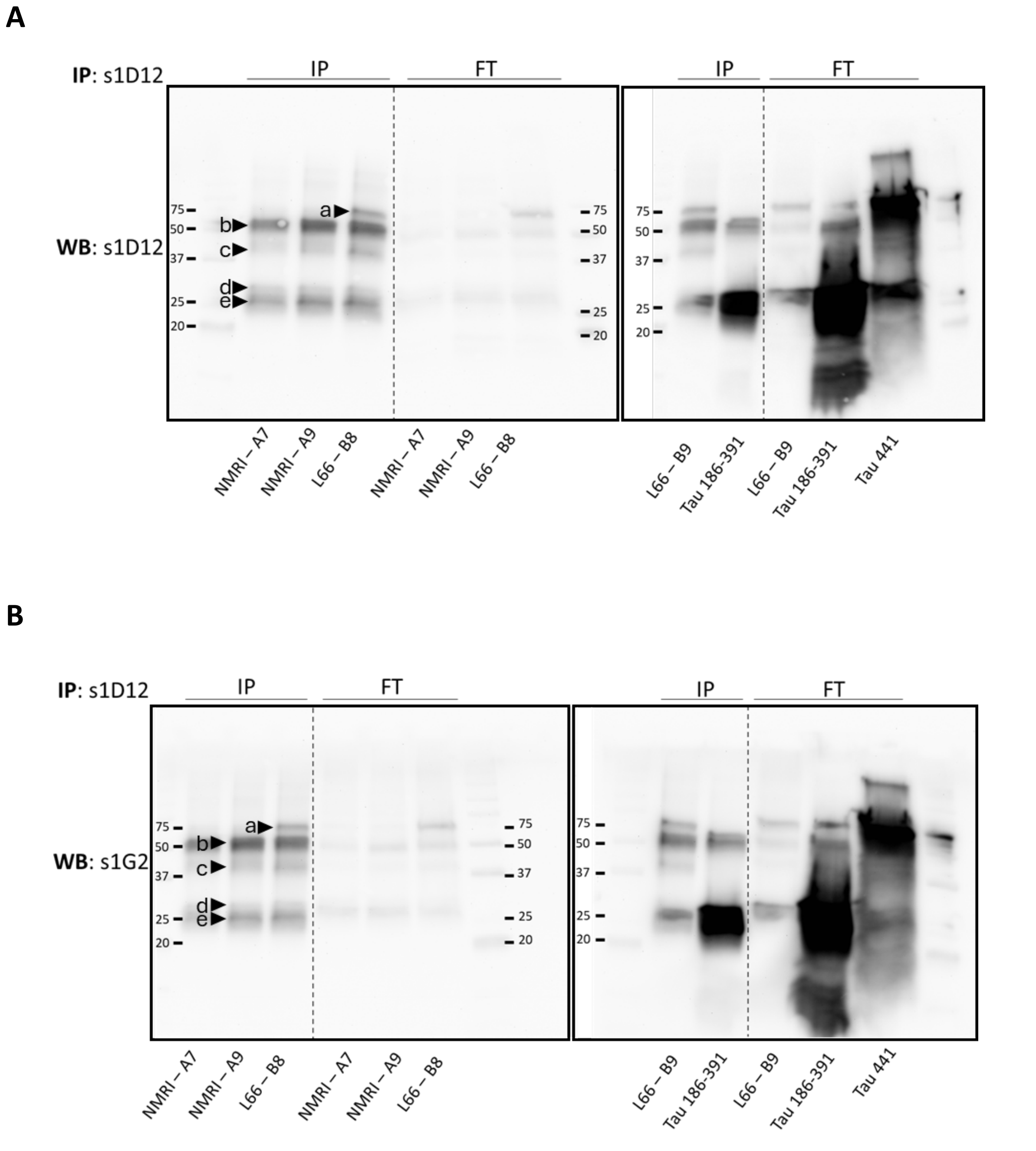

Figure S2: Verification of the efficacy of the immunoprecipitated tau samples further processed by MS/MS. Immunoblotting of immunoprecipitate (IP) and flow-through (FT) fractions using both s1D12 **(A)** and s1G2 **(B)** antibodies in L66$^{+/-}$ and NMRI mice. Recombinant Tau(186-391) and hT441 were used as positive controls. The presence of significant amount of both recombinant peptides (tau186-391 and hTau441) in the IP fractions is due to the fact that both were suspended in excess with the antibody-conjugated magnetic beads. Arrowheads: FL-hTau, a; FL-mTau, b; 36.3-kD band, c; 26.8-kD band, d; 24.5-kD band, e.

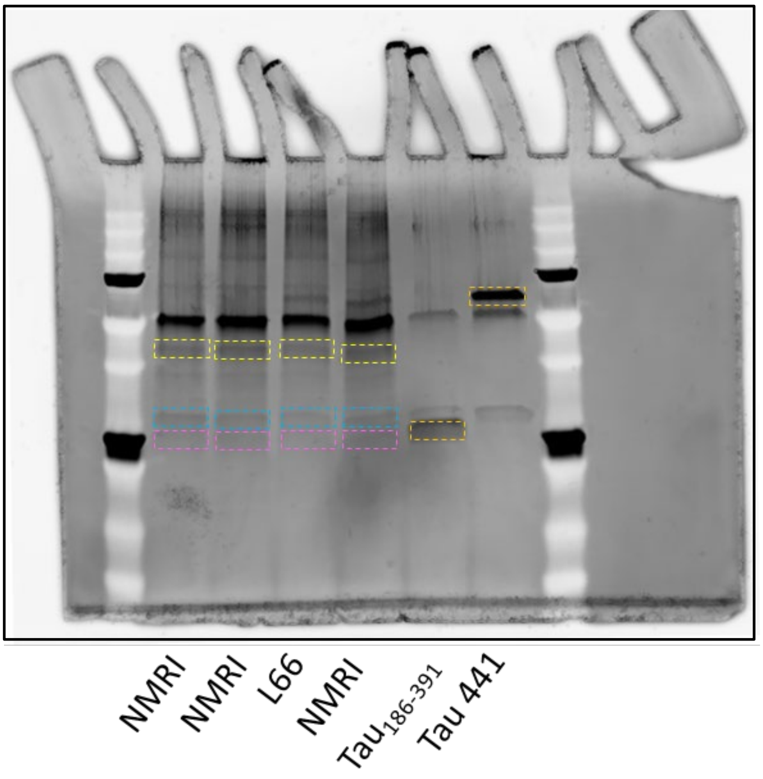

Figure S3: Total protein staining by SYPRO Ruby gel of the immunoprecipitated fractions in L66$^{+/-}$ and NMRI mice. Recombinant Tau(186-391) and hT441 were used as positive controls. The absence of smear in total protein staining indicates the efficacy of the immunoprecipitation in purifying the tau fragments also allowing a reliable excision of the bands to be further processed for the MS experiments. Coloured dashed rectangles: 36.3-kD bands, yellow; 26.8-kD bands, blue; 24.5-kD bands, pink; recombinant peptides, orange.

Table S1: Raw mass spectrometry data. There was a total of 166 protein hits likely present in the samples based on peptide match search by Mascot of a database consisting of consisted of 37,525 human and mouse protein sequences downloaded from UniProtKB (access date 30/11/2022) and including 8 human tau isoforms (fetal, A-G) and 5 mouse tau isoforms (A-E). Amino acids, AA; molecular weight, MW; calculated protein isoelectric point, calc. pI; peptide sequence matching, PSM. The longest human tau isoform (2N4R; Accession number: P10636-8) was used as reference for the sequence mapping of the detected peptides.
